# Temporarily induced facial eczema by IL-17 inhibitors: a case report and literature review

**DOI:** 10.3389/falgy.2025.1672897

**Published:** 2025-11-11

**Authors:** Ting Zhang, Fanzhang Meng, Junchen He, Chen Li, Zuotao Zhao

**Affiliations:** 1 Department of Dermatology, Tianjin Institute of Integrative Dermatology, Tianjin Academy of Traditional Chinese Medicine Affiliated Hospital, Tianjin, China; 2 School of Clinical Medicine, Beijing University of Chinese Medicine, Beijing, China

**Keywords:** IL-17 inhibitors, case report, psoriasis, eczema, paradoxical reactions

## Abstract

Biologics targeting interleukin-17 (IL-17) are widely used for moderate to severe psoriasis with great efficiency. Nonetheless, their usage has sporadically resulted in paradoxical reactions, such as eczema, sarcoidosis-like eruptions, alopecia areata, and pyoderma gangrenosum. Here, we report a case of temporary facial eczema to secukinumab with a score of 5 on the Naranjo scale, which suggests a probable drug side effect. The patient was a 32-year-old Chinese male with a history of chronic plaque psoriasis for 5 years. He was previously treated with topical steroids, calcipotriol, narrowband ultraviolet B phototherapy, and oral traditional Chinese medicine intermittently since 2020. In January of 2025, his psoriasis exacerbated and was not well controlled. The patient underwent an initial regimen of 300 mg secukinumab once weekly for 4 weeks, with significant psoriasis area and severity index (PASI) improvement, and was scheduled to continue maintenance therapy on a regimen of every 4 weeks. However, in the seventh week of the secukinumab treatment course, the patient's face developed diffuse, swollen, erythematous patches that had almost coalesced into sheets. The surface is smooth, without scales, blisters, or exudation, and accompanied by mild itching. Lab tests show elevated alanine aminotransferase (ALT) at 83.2 U/L (normal range: 9–50 U/L), slightly increased direct bilirubin at 8.48 μmol/L (normal range: 0–8.0 μmol/L). Other lab tests showed no significant abnormalities. After oral compound glycyrrhizin, olopatadine hydrochloride, triprolidine hydrochloride, and topical pimecrolimus for a week, his facial lesions were completely cleared. Liver function tests normalized following a 2-week course of polyenphosphatidylcholine. The patient delayed secukinumab administration by 2 weeks and continued 300 mg secukinumab administration on a regimen of every 4 weeks. No recurrence of similar rash or other adverse effects was observed during the subsequent follow-up period over 5 months. It is concluded that eczema could be induced temporarily by secukinumab, and maybe continued application.

## Introduction

Interleukin-17 (IL-17), first identified in 1993, represents a critical proinflammatory cytokine primarily secreted by Th17 cells, CD8+ T cells, γδ T cells, and natural killer T cells (NK cells). Non-IL-17 cells, such as mast cells and neutrophils, are also a source of IL-17. Mast cells are identified as major IL-17 producers in early acne lesions, particularly in CD4+ T-cell-rich areas, highlighting a critical IL-17/mast cell/T-helper cell axis in inflammatory skin conditions (1). In Alzheimer's disease, a specific subset of neutrophils producing IL-17 was identified (2). Mucosal-associated invariant (MAIT) cells produce IL-17A and IL-17F independently of IL-23, contributing to tissue inflammation in certain diseases (3). In psoriasis, γδ T cells and NK cells exhibit distinct but interconnected contributions to disease pathogenesis. γδ T cells (particularly Vγ4/Vδ1 subsets) are major contributors to psoriatic inflammation. They are among the highest producers of IL-17A and exhibit memory-like characteristics that mediate repeated inflammatory episodes (4). γδ T cells can suppress NK-cell responses through downregulation of NKG2D/NKp44 receptors (5) and show stronger immunological correlations with psoriasis progression than NK cells (6). NK cells may play more regulatory roles through interactions with γδ T cells rather than direct pathogenic effects (5).

The IL-17 family comprises six members (IL-17A-F) (7, 8). Under physiological conditions, IL-17A and related isoforms maintain immune homeostasis by combating pathogen invasion and mitigating bacterial infections. However, excessive IL-17 production has been implicated in chronic inflammatory processes, contributing to tissue damage and various autoimmune disorders, including psoriasis, spondyloarthropathies, rheumatoid arthritis, and multiple sclerosis (8). The therapeutic potential of IL-17 pathway inhibition (via cytokine/receptor-targeted biologics) has been well established in managing immune-mediated diseases. Several IL-17 inhibitors, such as secukinumab, ixekizumab, brodalumab, and bimekizumab, have received FDA approval. The main mechanism of action of secukinumab and ixekizumab is to inhibit IL-17A directly, whereas brodalumab inhibits the IL-17A receptor and bimekizumab inhibits IL-17A and IL-17F (9, 10). They are approved for the treatment of psoriasis, psoriatic arthritis, and ankylosing spondylitis.

While clinical trials demonstrate therapeutic efficacy, besides conventional adverse effects including allergy, infections, and hepatic dysfunction, other side effects such as paradoxical inflammatory responses also need to be considered. Several cutaneous paradoxical reactions have been reported in association with IL-17 inhibitors, such as eczema, sarcoidosis-like eruptions, alopecia areata (AA), pyoderma gangrenosum (PG), vitiligo, bullous pemphigoid, and pemphigus vulgaris. In this report, we present a case of temporary facial eczema probably induced by secukinumab for the treatment of plaque psoriasis. The patient’s facial eczema was successfully treated, and secukinumab administration was delayed by 2 weeks before being resumed at regular application. No recurrence of similar rash with 5 months of follow-up.

## Case report

**Figure 1:**
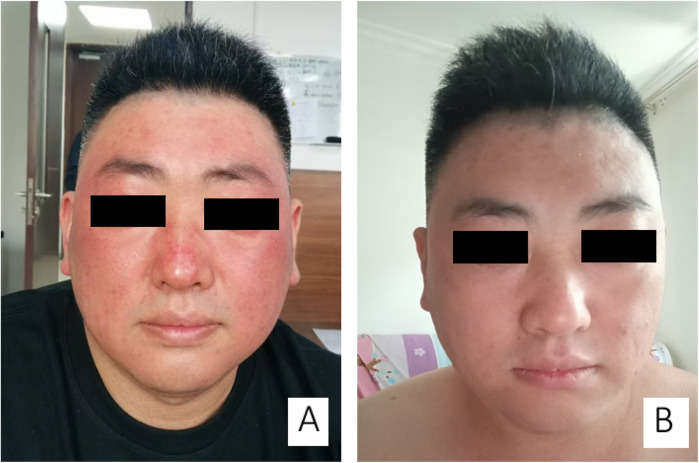
Two side-by-side images, labeled **(A)** and **(B)**, show a person with black bars covering their eyes. In image **(A)**, the person's face appears red and swollen. In image **(B)**, the redness and swelling have significantly reduced, and the skin appears clearer.

The patient was a 32-year-old Chinese male with a history of chronic plaque psoriasis for 5 years. He had no history of pustular psoriasis, erythrodermic psoriasis, or psoriatic arthritis during 5 years and no significant past medical history. The initial physical examination demonstrated hyperkeratotic red scaly plaques on the scalp, trunk, and limbs. He was previously treated with topical steroids, calcipotriol, narrowband ultraviolet B phototherapy, and oral traditional Chinese medicine intermittently since 2020. His rash has improved but still recurs. In January of 2025, his psoriasis exacerbated and was no longer well controlled, with approximately 25% body surface area affected. He started secukinumab 300 mg subcutaneous injection once weekly for 4 weeks, with significant psoriasis area and severity index (PASI) improvement, and was scheduled to continue maintenance therapy on a regimen of every 4 weeks. However, in the seventh week of the secukinumab treatment course, the patient's face developed diffuse, swollen, erythematous patches that had almost coalesced into sheets. The surface was smooth, without scales, blisters, or exudation, and was accompanied by mild itching (Figure 1A). The patient had no prior history of eczema and no exposure to topical products, including sunscreens, cosmetics, or new detergents, nor did he ingest any photosensitizing foods or medications before and during the 7-week secukinumab treatment.

Physical examination: temperature 36.9°C, heart rate (HR) 72 bpm, respiratory rate (RR) 18, blood pressure (BP) 122/72 mmHg. Liver function tests revealed elevated alanine aminotransferase (ALT) at 83.2 U/L (normal range: 9–50 U/L) and slightly increased direct bilirubin at 8.48 μmol/L (normal range: 0–8.0 μmol/L). Other laboratory tests, such as complete blood count (CBC), erythrocyte sedimentation rate (ESR), renal function, electrolytes, rheumatoid factor, total IgE, hepatitis B, hepatitis C, syphilis, HIV, urinalysis, and stool routine, showed no significant abnormalities. Electrocardiogram and chest x-ray showed normal findings. The patient reported normal liver function test results prior to initiating secukinumab therapy. He was diagnosed with facial eczema due to secukinumab with a score of 5 on the Naranjo scale suggesting a probable drug side effect, and hepatic abnormality was recognized as being induced by secukinumab. He received a prescription including oral compound glycyrrhizin, olopatadine hydrochloride, triprolidine hydrochloride, topical pimecrolimus for eczema treatment, and polyene phosphatidylcholine for liver protection therapy. The skin lesions resolved completely 1 week after treatment (Figure 1B). Transaminases and bilirubin normalized after 2 weeks of therapy. The patient delayed secukinumab administration by 2 weeks and continued 300 mg secukinumab subcutaneous injection on a regimen of every 4 weeks. No recurrence of similar rash or other adverse effects was observed during the subsequent follow-up period over 5 months.

## Discussion

IL-17 exhibits a wide range of biological functions, demonstrating both protective and detrimental roles in various physiological and pathological processes (11). It has complex roles in cancer and inflammation, acting as both a promoter and suppressor depending on the tumor microenvironment (TME) (12). It also plays a crucial role in protective immunity against extracellular pathogens (13). All biologics carry infection risks, although comparative studies show IL-17 inhibitors may have higher infection rates than IL-23 inhibitors (14). Malignancy risk is a concern for long-term treatment with both IL-17 and IL-23 inhibitors (15). Similar to other biologics, IL-17 inhibitors show increased short-term risks of nasopharyngitis and upper respiratory tract infections. Secondary treatment failure occurs with both tumor necrosis factor (TNF) inhibitors and IL-17 inhibitors, necessitating alternative therapies (16). IL-17 inhibitors are specifically associated with higher candidiasis risk compared with other biologics, particularly vs. IL-23 inhibitors (17), and demonstrated superior efficacy in hidradenitis suppurativa (HS) compared with IL-23 inhibitors which showed inconsistent results (18). First-generation anti-IL17 drugs showed an unexpected lack of efficacy in inflammatory bowel disease (IBD), unlike other biologics (19). The literature suggests that while IL-17 inhibitors share many class-wide biologic risks, their specific mechanism leads to distinct efficacy patterns (strong in HS/psoriasis but not IBD) and unique safety considerations (candidiasis risk) (18).

Here, we focus on discussing cutaneous paradoxical reactions of IL-17 inhibitors. We have summarized the clinical information regarding paradoxical skin reactions possibly induced by IL-17 inhibitors before August 2025 in PubMed and selected some cases in each paradoxical reaction shown in Table 1. Cutaneous paradoxical manifestations are diverse, with atopic dermatitis (AD) as the most common. Initial reports by Hattori et al. (20) documented AD exacerbation in a psoriasis patient receiving secukinumab. The paradoxical eczematous response triggered by IL-17 inhibitor biologics has been well documented with various cases published (21–23). Subsequent studies revealed *de novo* AD development in ankylosing spondylitis (AS) (24) and psoriasis populations. In a meta-analysis, PubMed, Embase, Cochrane Library, and Web of Science databases were searched for studies published before 1 March 2022 that reported at least one adverse event (AE). Dichotomous variables and 95% confidence intervals (CI) were analyzed using R software (version 4.1.3). Eczema was reported in 67 out of 1,230 patients who received IL-17 inhibitor biologics (25). Upon discontinuing biologics and switching to oral cyclosporine, antihistamines, Janus kinase 1 inhibitor, and topical glucocorticoids, patients experienced significant improvement in their skin lesions.

**Table 1:** Reported patients’ characteristics probably induced by IL-17 inhibitor-associated cutaneous paradoxical manifestations.

Cutaneous paradoxical manifestations	Age (years)	Sex	Previous history	Biologics	Interval time	Clinical outcome	Reference
AD/eczema	14 20	Male Male	Psoriasis Psoriasis	Secukinumab	8–12 weeks	Discontinue IL-17 inhibitors, low-dose prednisone, upadacitinib, cyclosporine	Brazen et al. (21) Xiao et al. (23)
Alopecia areata	64 70 40	Female Male female	Psoriasis Psoriasis Psoriasis	Secukinumab Ixekizumab Brodalumab	6–8 weeks 13 months 8 weeks	Discontinue IL-17 inhibitors, JAK inhibitors	Choi et al. (34) Eldirany et al. (35) Yajima et al. (36)
Pyoderma gangrenosum	68 61 43	Male Female Female	Psoriasis Psoriasis Psoriasis/HS	Secukinumab Ixekizumab Brodalumab	3 years 3 months 4 months	Discontinue IL-17 inhibitors, corticosteroids, cyclosporine, or JAK inhibitors	Orita et al. (40) Pollack et al. (41) Salvia et al.
Vitiligo	30 71	Male Male	Psoriasis Psoriasis	Secukinumab Ixekizumab	9 months 8 weeks	Discontinue IL-17 inhibitors or JAK inhibitors	Bouzid et al. (43) Eker et al. (44)
Bullous pemphigoid	66 77	Male Male	Psoriasis Pityriasis rubra pilaris	Secukinumab Ixekizumab	5 weeks 38 weeks	Discontinue IL-17 inhibitors, systemic corticosteroids, doxycycline	Wang et al. (46) Sugihara et al. (47)
Pemphigus vulgaris	72	Female	Psoriasis	Secukinumab	4 weeks	Discontinue IL-17 inhibitors, corticosteroids, rituximab (anti-CD20) immunosuppressants	Minai et al. (48)
Granuloma annulare	60	Female	Psoriasis/psoriatic arthritis	Secukinumab	2 weeks	Discontinue IL-17 inhibitors, corticosteroids, antibiotics, etanercept	Bonomo et al. (49)
Hidradenitis suppurativa	58	Male	Psoriasis/psoriatic arthritis	Secukinumab	4 weeks	Discontinue IL-17 inhibitors, ustekinumab, adalimumab	Navarro-Triviño et al. (50)
Acquired ichthyosis	72	Male	Psoriasis	Brodalumab	3 months	Discontinue IL-17 inhibitors	Mantovani et al. (52)

In this case, the patient started subcutaneous injection of 300 mg secukinumab once weekly for 4 weeks and was scheduled to continue maintenance therapy on a regimen of every 4 weeks. Unfortunately, his facial eczema developed in the seventh week of the secukinumab treatment course. The patient's intensive initial treatment over the first 4 weeks likely resulted in relatively high drug concentrations, which may have triggered an immune response in the body. After the eczema resolved with treatment, the maintenance therapy was deliberately delayed by 2 weeks but still administered every 4 weeks as a standard dosing regimen. In the reported literature, paradoxical reactions following secukinumab administration typically can lead to discontinuation of the drug. However, in this case, after careful consideration, we just delayed the first maintenance dose by 2 weeks but continued the treatment according to the original schedule. This decision was based on the mild and reversible nature of the adverse reaction, which could be managed with symptomatic treatment. We also took into account the established long-term safety profile of secukinumab (26, 27). During the subsequent 5 months of follow-up, no recurrence of a similar rash was observed. This absence of recurrence is possibly related to the comparatively lower drug concentrations during this phase. The patient's rash was localized to the facial area. We have ruled out potential local contact triggers or photosensitivity. The patient did not use sunscreen, cosmetics, or any other topical products before or during secukinumab treatment, nor did he ingest any photosensitizing foods or medications. A review of the literature found no direct evidence indicating that secukinumab increases photosensitivity. Studies suggest that biological agents, including IL-17 inhibitors, may induce eczematous rashes through abnormal immunomodulation in the skin, presenting as skin dehydration or delayed hypersensitivity reactions (28). Additionally, inhibition of IL-17A—which plays a role in mucosal defense—may compromise skin barrier function and increase the risk of eczema (29), particularly in sensitive areas such as the face. We hypothesize that this mechanism may be relevant in the present case.

Psoriasis and eczema are characterized by an imbalance in the T-helper (Th)1/Th2 immune response, Th1/Th2 immune pathways are closely related, such that when the Th1 response is blocked or decreased, Th2 increases to maintain balance. It is plausible that, as IL-17 inhibitor therapy decreases Th1, there is subsequent shunting to a Th2-dominant immune response, thereby resulting in an eczematous reaction (30). In contrast, Al-Janabi et al. (31) found increased expression of TNF, interferon (IFN)-γ, and IFN-α and their signaling pathways in paradoxical eczema case cell clusters compared with those in matched psoriasis controls. This suggests that paradoxical eczema has a predominantly type 1 systemic inflammatory signature and that genetic susceptibility to aberrant chemokine and TNF pathway signaling could contribute to the development of this phenotype during biologic treatment. Serum proteomic analysis revealed that patients with paradoxical eczema already exhibit AD-like inflammatory signatures even before the clinical phenotype manifests, evidenced by enrichment of gene sets related to Th2 cytokines such as IL-4 and IL-13 (32). However, the local skin microenvironment demonstrates upregulation of Th1/Th17 pathways (e.g., elevated IL-17A mRNA), consistent with psoriasiform or lichenoid dermatitis (33).

Alopecia areata is probably induced following secukinumab (34), ixekizumab (35), and brodalumab (36) administration in psoriasis, palmar and plantar pustula psoriasis, or psoriatic arthritis (PsA) patients. Interleukin 17 inhibitors were hoped to be a potential option for treating alopecia. However, a recent double-blinded, randomized clinical trial showed no significant benefit for treating AA with secukinumab (37). Therapeutic responses to corticosteroid regimens or drug discontinuation have been documented. The underlying mechanism of alopecia areata upon IL-17A inhibitor exposure remains to be elucidated. The correlation between PG and IL-17 inhibitors involves complex immunological mechanisms and clinical observations. PG is characterized by a profound dysregulation of both innate and adaptive immunity, with evidence pointing to the involvement of proinflammatory cytokines including IL-17, IL-23, and IL-1 family members (38). IL-17 is a key cytokine in neutrophilic inflammation, and its overproduction is implicated in PG pathogenesis. IL-17 promotes neutrophil recruitment and activation, which are central to the formation of PG's characteristic ulcers (39). Orita et al. (40) reported a case probably induced by PG by secukinumab. A 61-year-old woman with psoriasis described PG after ixekizumab administration (41). This paradoxical reaction suggests that IL-17 inhibition may disrupt immune homeostasis, potentially triggering PG in susceptible individuals. IL-17 inhibitors may paradoxically induce PG in rare cases, while targeting the IL-17/IL-23 axis or downstream pathways (e.g., JAK/STAT) can be therapeutic. The exact mechanisms remain under investigation, but dysregulated neutrophilic inflammation driven by IL-17 is a central feature (42). Combination therapy with immunosuppressants and biologics demonstrates clinical efficacy.

New-onset vitiligo in PsO/PsA patients may be associated with secukinumab (43) and ixekizumab (44). Although circulating IL-17 levels and T-helper type 17(Th17) numbers have been shown to increase in vitiligo patients, the pathogenic role of IL-17 and Th17 in vitiligo is debatable. Scientists found that patients with progression of vitiligo have signiﬁcantly increased Th17.1 and Th1 lymphocytes, but not Th17 lymphocytes (45). Postulate that targeting IL-17 inhibits Th17, skewing toward a predominantly Th1 response that exacerbates vitiligo. On the contrary, broader immunosuppressive properties, such as cyclosporine, JAK inhibitors, and methotrexate, do not inﬂuence the Th1/Th17 balance and therefore control both psoriasis and vitiligo. Bullous pemphigoid induction was demonstrated by initiation of secukinumab (46) and ixekizumab (47). This might be associated with a helper T-cell Th1/Th2 imbalance. Steroid–antibiotic combinations effectively resolve lesions. Another case presents a patient who suffered pemphigus vegetans and showed an exacerbation of pemphigus foliaceus after secukinumab loading for the treatment of complicated generalized pustular psoriasis and pyoderma gangrenosum (48). The emergence of granuloma annulare (GA) after beginning IL-17 inhibitors was reported (49). Additionally, a case of HS was diagnosed after secukinumab treatment; the patient was subsequently switched to ustekinumab to manage dermatological symptoms, rhematological manifestations, and HS (50). Another case transferred to adalimumab to improve HS skin lesions successfully (51). A unique case of ichthyosis possibly induced by brodalumab prescription was resolved with emollient therapy (52).

While IL-17 inhibitors demonstrate significant therapeutic value, clinicians must remain vigilant for paradoxical inflammatory responses. Mechanistic understanding of immune axis dysregulation and tailored management strategies remain crucial in optimizing treatment outcomes. Continued pharmacovigilance and translational research are warranted given the expanding clinical applications of IL-17 inhibitors. Prior case reports indicate that paradoxical eczema typically presents with non-facial involvement, and its occurrence usually necessitates discontinuation of IL-17 inhibitors and switching to alternative biologic agents. However, our case is distinctive in several aspects: the rash emerged at week 7 following the 4-week intensive induction therapy; it was transient and confined exclusively to the facial region. After a deliberate 2-week treatment delay, therapy was resumed at the standard 4-week maintenance interval. Throughout the subsequent 5-month follow-up, no recurrence of the rash or other adverse effects was observed. Of course, continuous longer-term follow-up and monitoring are required. This outcome suggests that when mild and reversible paradoxical reactions occur during biologic therapy, clinicians may consider treatment continuation with dose interval adjustment rather than immediate discontinuation, following a careful risk–benefit assessment.

## Data Availability

The original contributions presented in the study are included in the article/Supplementary Material; further inquiries can be directed to the corresponding author/s.
